# Frontiers and hotspots in comorbid epilepsy and depression: a bibliometric analysis from 2003 to 2023

**DOI:** 10.3389/fneur.2024.1413582

**Published:** 2024-06-21

**Authors:** Gui-Yu Liu, Fan-Jia Fu, Ying-Xin Chou, Ming-Sha Ye, Yi-Lin Ouyang, Ming-De Yan, Lei Pan, Wei-Peng Li, Wei Xie

**Affiliations:** ^1^School of Traditional Chinese Medicine, Southern Medical University, Guangzhou, China; ^2^Department of Neurology, Hospital of Integrated Traditional Chinese and Western Medicine, Southern Medical University, Guangzhou, China; ^3^Department of Traditional Chinese Medicine, Nanfang Hospital, Southern Medical University, Guangzhou, China

**Keywords:** CiteSpace, bibliometrics, comorbid epilepsy and depression, epilepsy, depression, VOSviewer

## Abstract

**Background:**

Epilepsy ranks among the most common neurological disorders worldwide, frequently accompanied by depression as a prominent comorbidity. This study employs bibliometric analysis to reveal the research of comorbid epilepsy and depression over the past two decades, aiming to explore trends and contribute insights to ongoing investigations.

**Methods:**

We conducted a comprehensive search on the Web of Science Core Collection database and downloaded relevant publications on comorbid epilepsy and depression published from 2003 to 2023. VOSviewer and CiteSpace were mainly used to analyze the authors, institutions, countries, publishing journals, reference co-citation patterns, keyword co-occurrence, keyword clustering, and other aspects to construct a knowledge atlas.

**Results:**

A total of 5,586 publications related to comorbid epilepsy and depression were retrieved, with a general upward trend despite slight fluctuations in annual publications. Publications originated from 121 countries and 636 institutions, with a predominant focus on clinical research. The United States led in productivity (1,529 articles), while Melbourne University emerged as the most productive institution (135 articles). *EPILEPSY & BEHAVIOR* was the journal with the highest publication output (1,189 articles) and citation count. Keyword analysis highlighted emerging trends, including “recognitive impairment” and “mental health,” indicating potential future research hotspots and trends.

**Conclusion:**

This study is one of the first to perform a bibliometric analysis of the 20-year scientific output of comorbid epilepsy and depression. While research has trended upwards, ambiguity in pathogenesis and the absence of standardized diagnostic guidelines remain concerning. Our analysis offers valuable guidance for researchers, informing that this might be a strong area for future collaborations.

## 1 Introduction

Epilepsy is a prevalent neurological disorder characterized by recurrent seizures (1) that affects approximately 70 million of people worldwide (2, 3). Accumulating evidence emphasizes the intricate interactions between epilepsy and various mental disorders. Prolonged seizures in epilepsy can lead to complications such as depression, anxiety, dementia, asthma, and peptic ulcer disease (4), with depression being the most common comorbidity (5). Approximately one-third of people with epilepsy suffer from depression, significantly diminishing their quality of life (6) and social engagement due to the sudden unexpected seizures (7, 8). The reported prevalence of depression in people with epilepsy ranges from 10.7 to 44%, reaching up to 54% in refractory epilepsy cases (9). Conversely, meta-analyses reveal epilepsy prevalence rates ranging from 1 to 6% among individuals with depression. Therefore, the coexistence of epilepsy and depression demands significant attention due to its complexity and profound impact on patients’ lives.

To comprehensively understand the current research status and future development trend of comorbid epilepsy and depression, this study utilized the Web of Science Core Collection database and analyzed using bibliometric software such as CiteSpace and VOSviewer. A total of 5,586 publications related to comorbid epilepsy and depression from 2003 to 2023 were evaluated for their characteristics, including annual publication volume, countries, institutions, authors, keywords and references. The study aims to reveal the distribution of journals, identify core authors, trace the development trends of literature, and outline hotspots and frontiers in the research field of comorbid depression in epilepsy, providing valuable references for researchers in this domain.

## 2 Data and methods

### 2.1 Data source and search strategies

The data in this study were obtained from the Web of Science Core Collection (WoSCC). Using the “Advanced Search” function in the WoSCC database, the search query was set as Topic = (Epilepsy comorbid depression) OR Topic = (Epilepsy with depression) and Index = the Science Citation Index Expanded of Web of Science Core Collection (WoSCC). Through this query, 6,092 articles were obtained. Simultaneously, additional search conditions (Time Span = 2003/01/01–2023/07/01) were applied, and as of August 23, 2023, after eliminating multiple interference factors, a total of 5,586 articles were retrieved, including 4,561 articles and 1,025 review articles.

### 2.2 Inclusion criteria

Relevant literature in the field of comorbidity of epilepsy and depression.

### 2.3 Exclusion criteria

Exclusion of non-English literature, conference abstracts, conference documents, editorial materials, letters, preprints, book chapters, and materials unrelated to the field of comorbid depression in epilepsy.

### 2.4 Research methods and analysis tools

The publications obtained from the Web of Science Core Collection (WoSCC) search was exported in plain text format. CiteSpace, Bibliometrix and VOSviewer were employed for visual analysis of aspects such as annual publication volume, countries, institutions, authors, and keywords. Knowledge maps were generated, and the results were analyzed accordingly.

VOSviewer, developed by Nees Jan van Eck and Ludo Waltman at the Centre for Science and Technology Studies (CWTS) of Leiden University, is a JAVA-based software tool primarily used in this study for building and visualizing relationships in bibliographic data. It can generate various graph types based on bibliometric relationships, displaying the structure and collaboration relationships in a knowledge domain. CiteSpace, developed by Dr. Chaomei Chen, a professor of computer and information science at Drexel University, is a JAVA-based software tool for visualizing the structure, patterns, and distribution of scientific knowledge, It produces graphs called “knowledge maps” and is useful for tasks such as writing review papers and scanning hotspots in academic research. Bibliometrix is an R package designed for bibliometric analysis, effectively extracting and analyzing large-scale bibliographic data to help researchers explore trends in scientific research.

## 3 Results

### 3.1 Publication trends and journals

**Figure 1:**
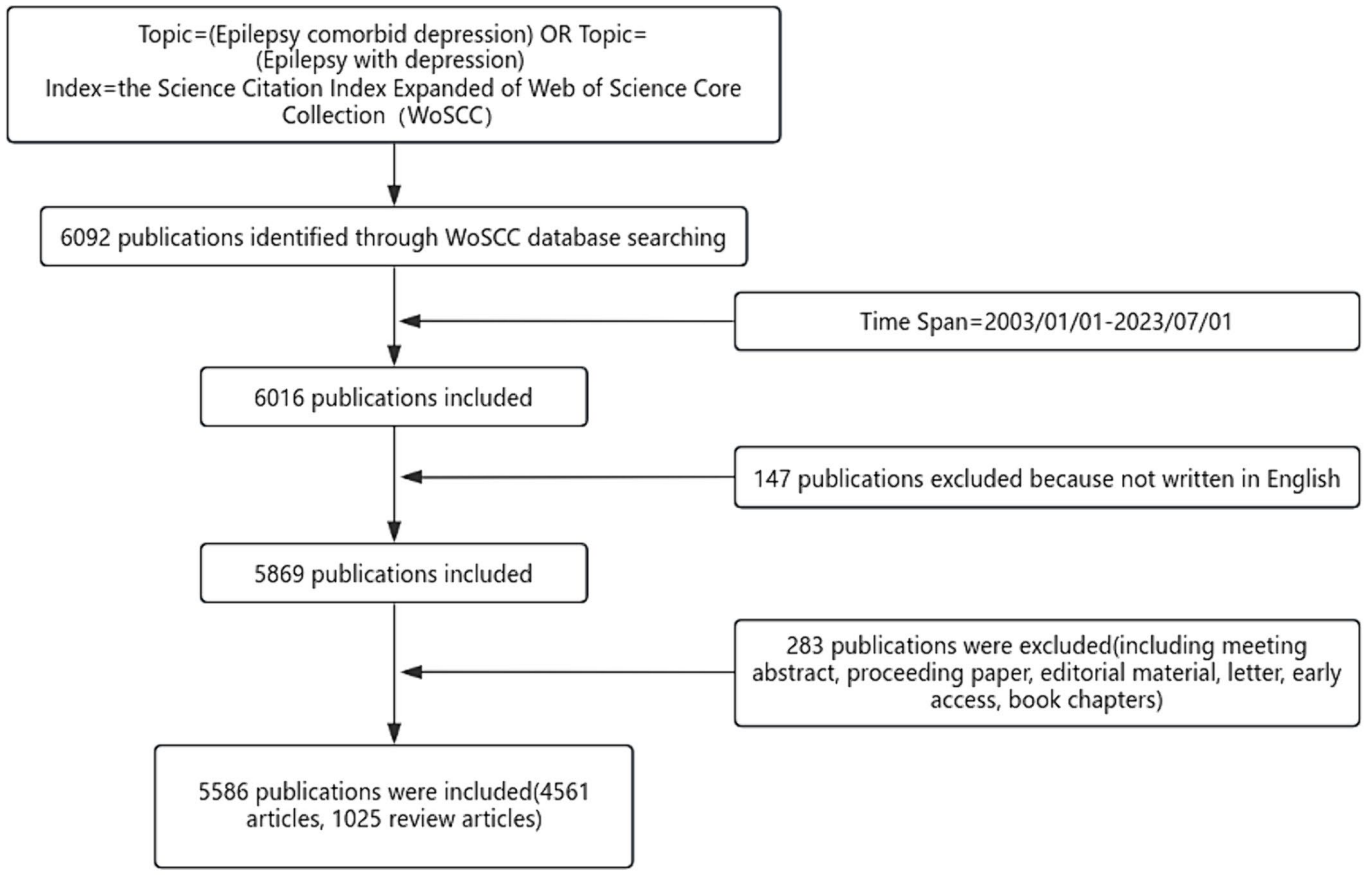
The flow chart of document collection and sorting.

**Figure 2:**
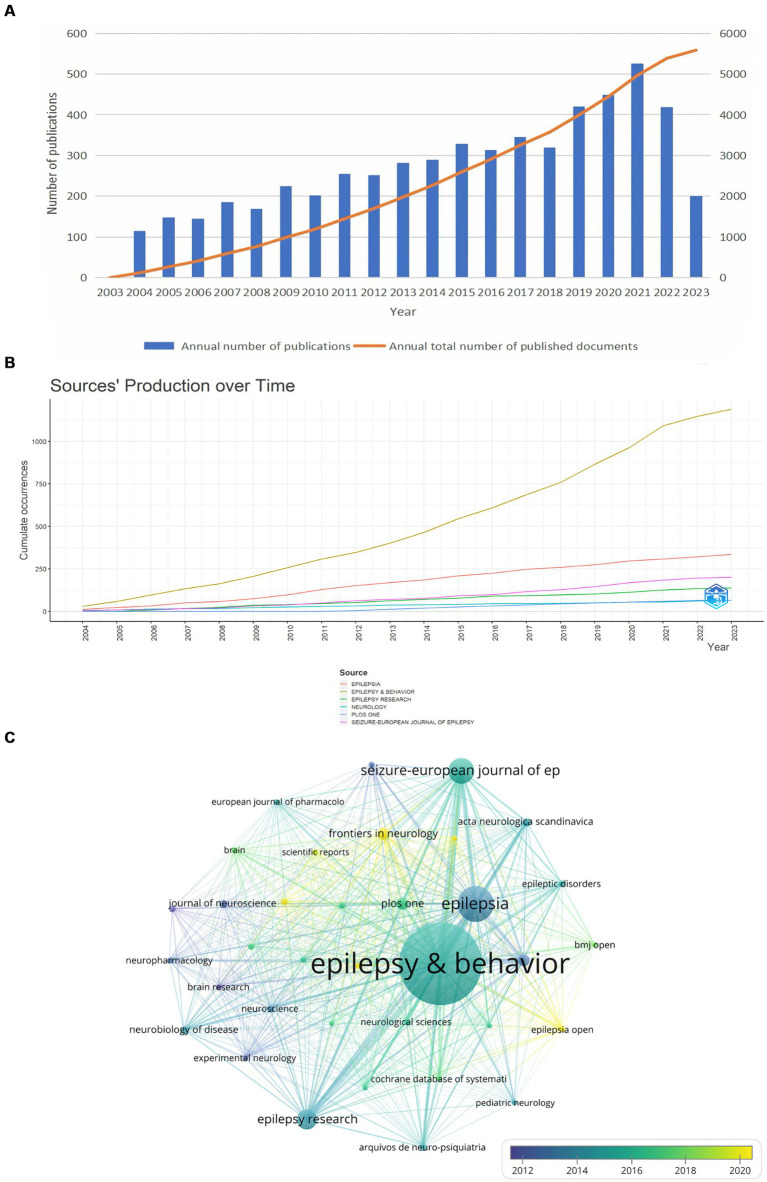
Analysis of articles related to epilepsy comorbidities and depression. **(A)** Annual trends in the number of publications for epilepsy comorbidities and depression. **(B)** Annual publications for the top six journals. **(C)** Journal citation frequency. The chart shows the journals with more than 20 citations. The node size is proportional to the number of references, and the color change within the node is related to the year, with blue indicating an earlier time and yellow indicating a time closer to the present.

Following the inclusion and exclusion criteria, a total of 5,586 pieces of related literature related to comorbid epilepsy and depression were included (Figure 1). Figure 2A shows that the annual number of publications demonstrates an overall upward trend, reaching a peak of 526 in 2021, which suggested that related studies of comorbid depression in epilepsy have attracted greater attention and may be a hotspot in current research.

Bibliometrix was utilized to visualize the annual publication trends of the top six journals in terms of publication volume (Figure 2B). Subsequently, we identified and screened a total of 35 relevant journals based on a minimum publication threshold equal to or greater than 20 articles each, resulting in the creation of a journal cooperative network using VOSviewer in Figure 2C.

We also verified that several key journals in Table 1, such as *EPILEPSY & BEHAVIOR* (1,189 articles), *EPILEPSIA* (336 articles), *SEIZURE-EUROPEAN JOURNAL OF EPILEPSY* (201 articles), *EPILEPSY RESEARCH* (138 articles), *NEUROLOGY* (66 articles), *PLOS ONE* (66 articles), which serve as crucial references in the study of comorbid depression in epilepsy. Furthermore, *EPILEPSY & BEHAVIOR* and *EPILEPSIA* journals had the highest average citation counts (Figure 2C).

**Table 1:** The top 15 countries/regions in terms of publication volume.

Sources	Articles
EPILEPSY & BEHAVIOR	1,189
EPILEPSIA	336
SEIZURE-EUROPEAN JOURNAL OF EPILEPSY	201
EPILEPSY RESEARCH	138
NEUROLOGY	66
PLOS ONE	66
FRONTIERS IN NEUROLOGY	64
JOURNAL OF NEUROSCIENCE	38
ACTA NEUROLOGICA SCANDINAVICA	37
NEUROBIOLOGY OF DISEASE	37
ARQUIVOS DE NEURO-PSIQUIATRIA	31
NEUROSCIENCE	31
EPILEPTIC DISORDERS	29
FRONTIERS IN NEUROSCIENCE	28
JOURNAL OF NEUROPHYSIOLOGY	27

### 3.2 Author visualization analysis

The analysis of author publication volume and author cluster distribution reveal the influential research groups and potential collaborative relationships.

**Figure 3:**
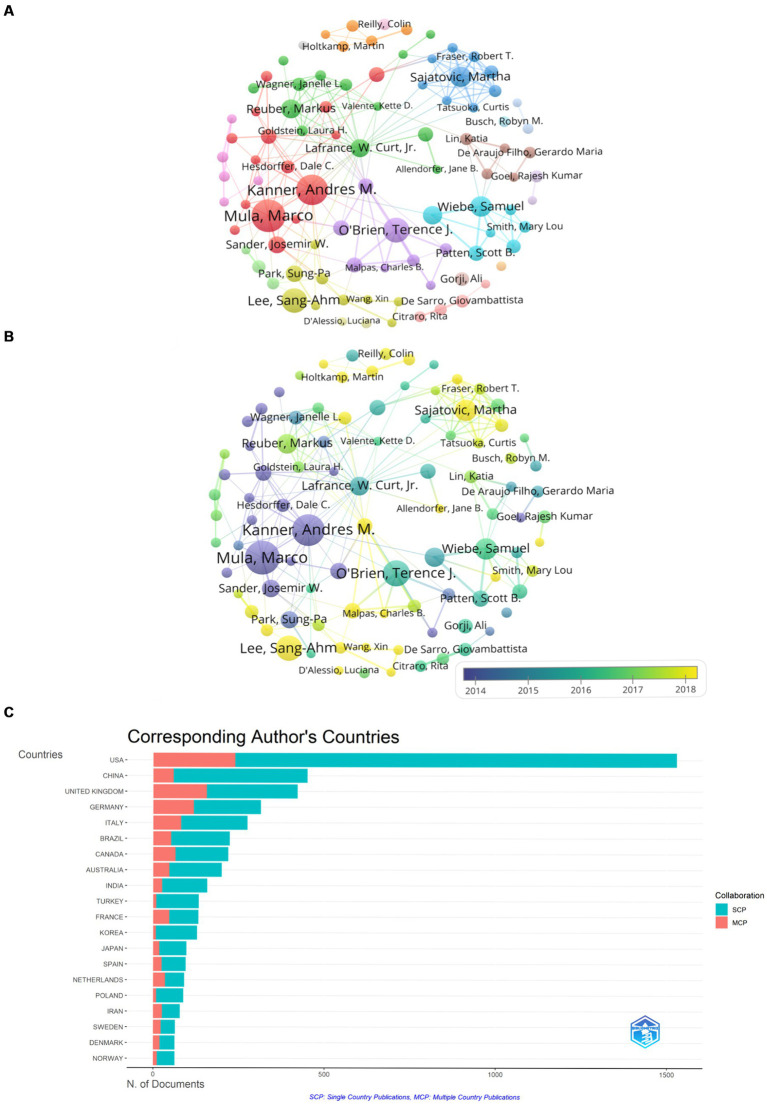
Visual analysis of the author. **(A)** Co-production by authors. Shows the abbreviated names of authors with more than 12 papers. Node size is proportional to the number of publications. **(B)** Author collaboration (time dependent). It shows the change in the number of papers published by 94 authors over time, and the color change in the node is related to the year, purple indicates the earlier activity time, while yellow indicates the activity time closer to the present. **(C)** Corresponding author country and cooperation. Red represents the number of articles co-authored with authors from other countries, and blue represents the number co-authored with authors from the same country.

Figure 3A presented 94 authors with publication volumes of 12 or more and their collaborative relationships. The top three authors in terms of publication volume were *Mula, Marco* (59 articles), *Kanner, Andres M.* (55 articles), and *O’Brien, Terence J.* (43 articles). Most of the nodes had multiple connections with several authors nearby at the same time, indicating the existence of teamwork and strong collaboration in the research of comorbid depression in epilepsy.

Figure 3B displayed the changes in publication volume for these 94 authors over time. It reveals that the substantial publications by teams led by *Mula, Marco,* and *Kanner, Andres M.* were mainly concentrated around 2014, laying the foundation for the study of comorbid epilepsy and depression. In recent years, smaller teams led by authors such as *Lee*, *Sang-Ahm*, and *Sajatovic, Martha* have contributed significantly, with publication volumes reaching 42 and 34 articles, respectively.

Figure 3C illustrated the nationality of corresponding authors and the international collaboration rate. It can be inferred that the United States has the highest publication volume (1,529 articles), contributing to 1,290 domestic collaborative publications and 239 international collaborative publications in literature related to comorbid epilepsy and depression. MCP represents the number of papers co-authored with authors from other countries. MCP ratio reflects the ratio of internationally collaborative publications to the total publications, providing insights into international collaboration. However, the MCP ratio for the United States is 0.156, indicating relatively low international collaboration. China follows with a total publication volume of 450 articles, including 391 domestic collaborative publications and 59 international collaborative publications, and the MCP ratio for China is 0.131, indicating that research in China primarily also focuses on domestic collaboration. The United Kingdom ranks third with a total publication volume of 421 articles, including 265 domestic collaborative publications and 156 international collaborative publications. The MCP ratio for the United Kingdom is 0.371, suggesting a closer collaboration with other countries compared to the United States and China.

### 3.3 Countries and institutions

**Figure 4:**
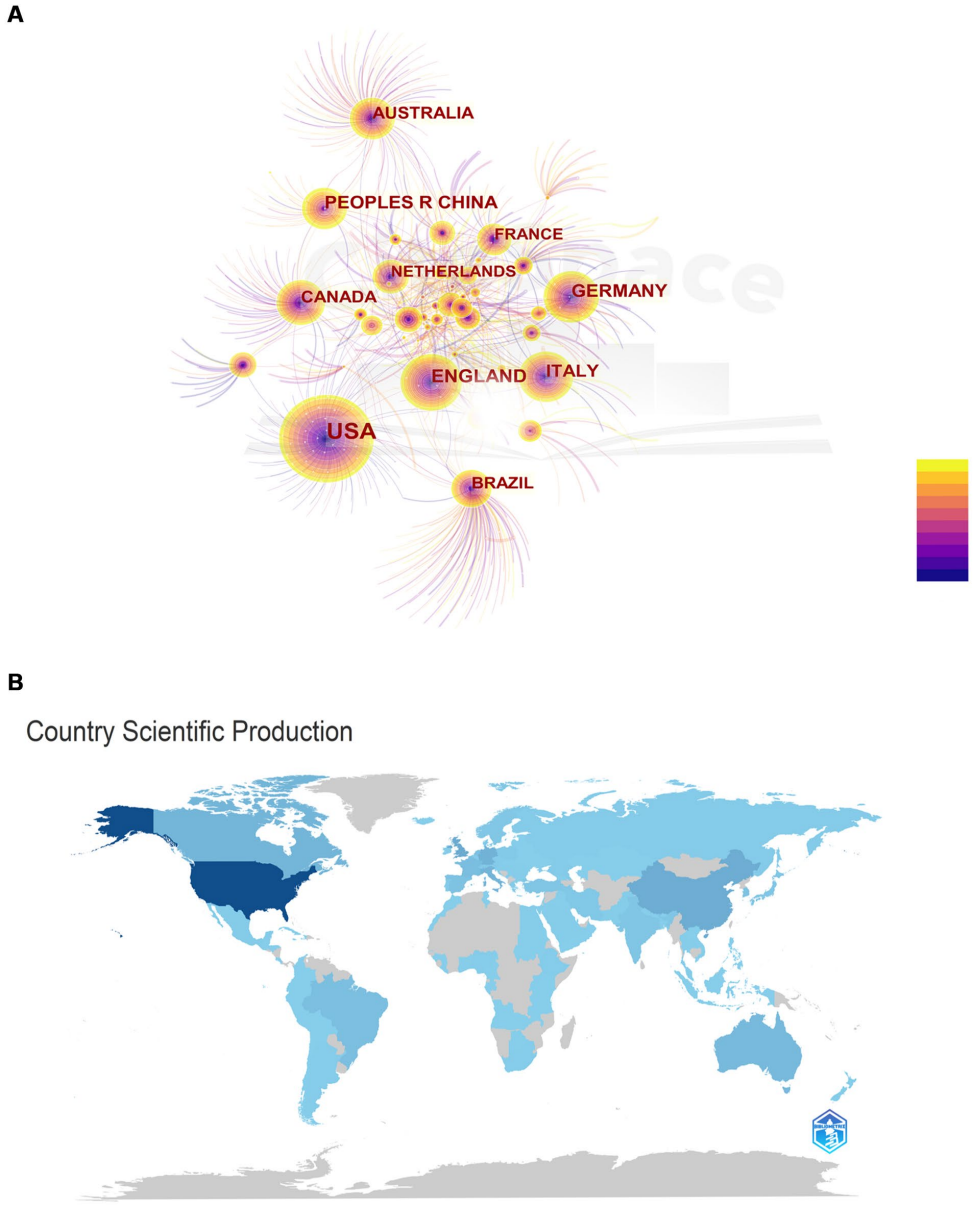
National contributions. **(A)** Networks of cooperation among States. The chart shows the names of the 121 countries in which publications were made. The node size is proportional to the number of publications, and the color variation within the node is related to the year of activity, with purple indicating an earlier time of activity and yellow indicating a time closer to the present. **(B)** Map of countries participating in research related to epilepsy comorbidities depression. The level of participation of the country is proportional to the depth of the color.

As shown in Figure 4, a total of 121 countries contributed to publications on comorbid epilepsy and depression. And the top 10 countries in terms of publication volume accounted for 83.17% of the total publications (4,646 out of 5,586), with the United States (1906, accounting for 34.12% of the total), the United Kingdom (620, 11.1%), China (453, 8.12%), Germany (444, 7.95%), Italy (387, 6.93%), Canada (333, 5.96%), Australia (280, 5.01%), Brazil (259, 4.63%), France (202, 3.61%), and Netherlands (198, 3.54%).

**Figure 5:**
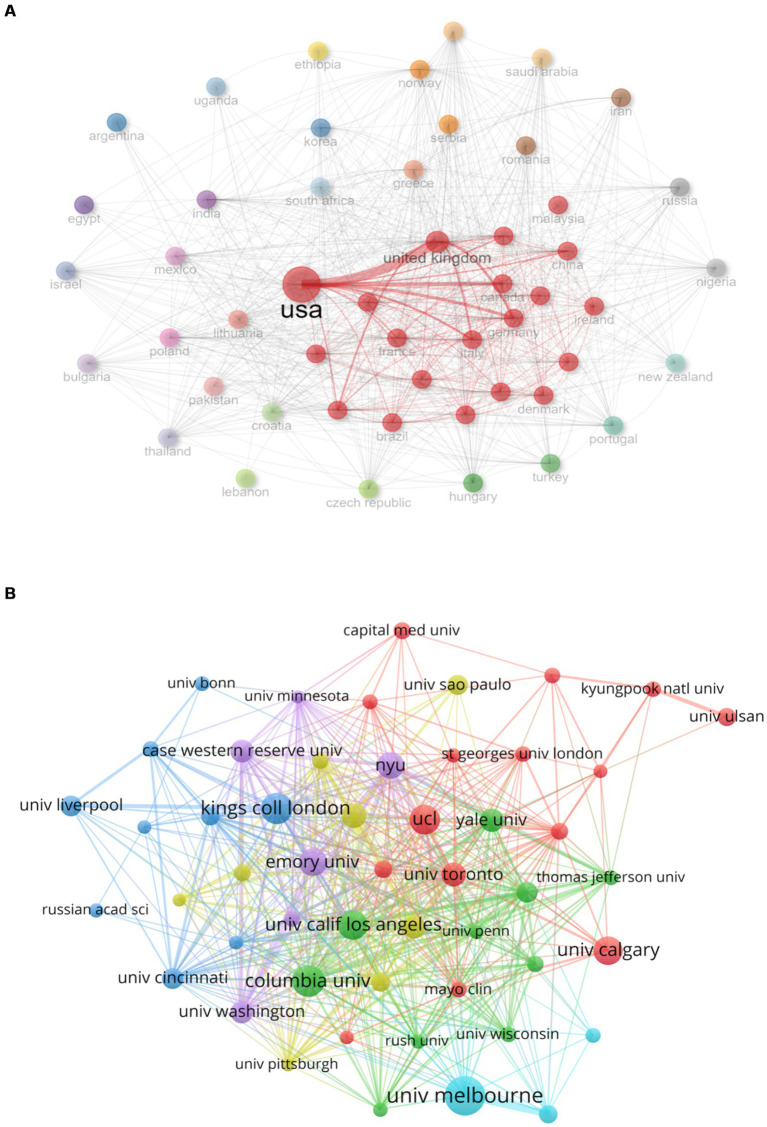
Analysis of national and institutional contributions. **(A)** National cooperation networks. The red circle represents countries with strong links to other countries, and the thicker the line, the closer the relationship between countries. **(B)** Issuing institutions co-appear on the network. The chart shows the names of institutions that have published more than 32 articles. Different colored clusters indicate the degree of cooperation between institutions, and the size of nodes is proportional to the number of publications.

The country collaboration network graph (Figure 5A) reveals that the United States and the United Kingdom have the closest collaboration among all countries. Notably, countries within the red circle, denoting close connections, predominantly comprise developed countries, with the exception of China. This observation suggested that researchers in developed countries exhibit proficiency in international collaboration, facilitating information exchange for more in-depth and widespread research. VOSviewer was employed for visual analysis, setting the minimum publication volume for each institution at 32 articles. The result displayed 51 nodes, indicating that 51 institutions had published at least 32 articles (Figure 5B). According to publication volume, the top 10 institutions are the University of Melbourne (135 articles), Columbia University (102 articles), King’s College London (98 articles), University College London (93 articles), University of California, Los Angeles (91 articles), University of Calgary (87 articles), Emory University (85 articles), New York University (80 articles), Harvard University (74 articles), and the University of Toronto (70 articles). Notably, the top 10 institutions by publication volume are predominantly located in the United States, the United Kingdom, Canada, and Australia, while other countries in the top 10 publication volume list did not have institutions ranking in the top 10, indicating that the 4 countries play a leading role in the academic development of the field.

### 3.4 Keyword co-occurrence analysis

**Figure 6:**
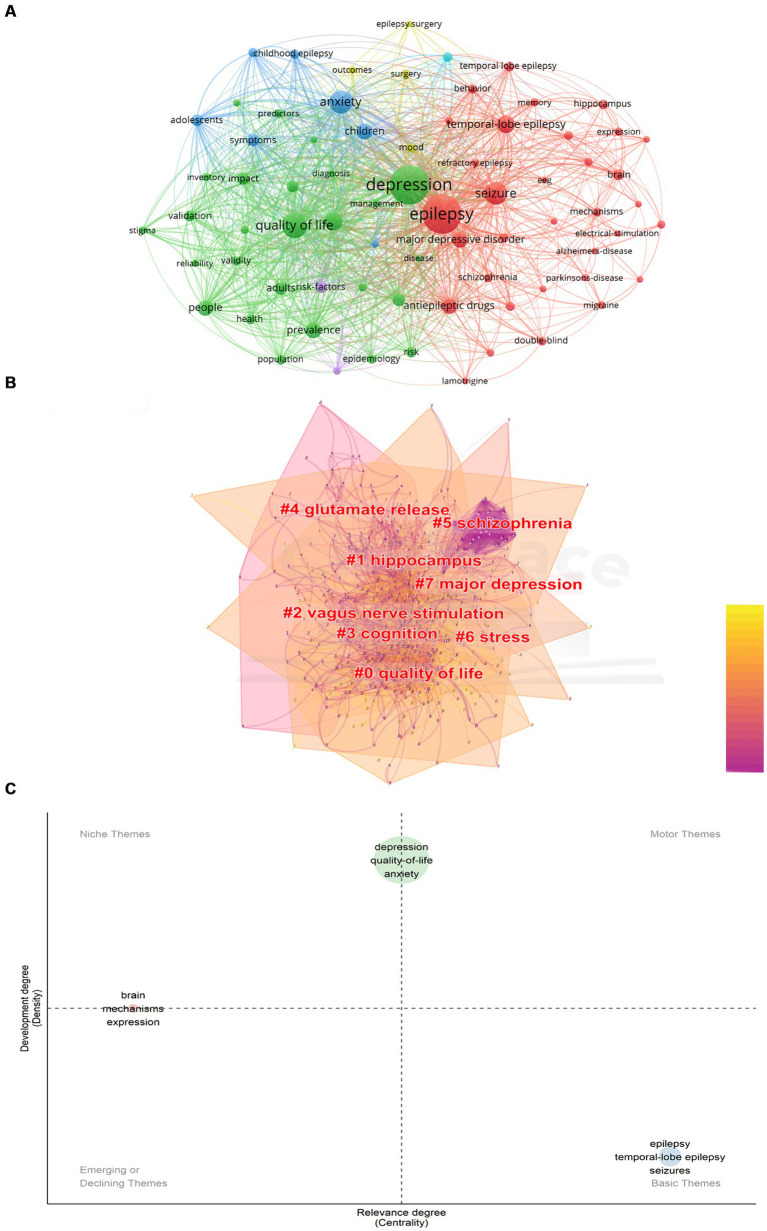
Keyword analysis. **(A)** Keyword co-occurrence network. The graph shows the names of keywords with frequencies greater than 100 times. The node size is proportional to the frequency of occurrence. **(B)** Keyword cluster analysis. The cluster name is marked in red, where the overlapping part of the color block indicates the overlap of the article classification. **(C)** Thematic map. Niche Themes indicate very professional but marginal Themes; Motor Themes indicate structural themes of well-developed and important research fields; Emerging or Declining Themes indicate themes that are just emerging or about to disappear. Basic Themes Indicates important but incomplete themes.

All collected literature data were imported into VOSviewer for analysis, merging different expressions of keywords with similar meanings to avoid potential deviations. The minimum frequency of keyword occurrences was set to 100 times, resulting in a visualized graph with 73 nodes (Figure 6A). Among these, “depression” had the highest frequency of 2,629 occurrences. High-frequency terms included “epilepsy” (2,614 occurrences), “quality of life” (1,198 occurrences), “anxiety” (970 occurrences), “seizure” (941 occurrences), “disorder” (637 occurrences), “temporal-lobe epilepsy” (626 occurrences), “children” (510 occurrences), “prevalence” (500 occurrences), “people” (479 occurrences), and “antiepileptic drugs” (469 occurrences).

To obtain a keyword clustering graph, CiteSpace was used for data analysis (Figure 6B). The keywords were primarily categorized into 8 groups (sequential order does not indicate priority): “quality of life,” “hippocampus,” “vagus nerve stimulation,” “cognition,” “glutamate release,” “schizophrenia,” “stress” and “major depression.” Overlapping color blocks indicated overlapping categories among articles.

Figure 6C showed various thematic categories: “Niche Themes” representing highly specialized but somewhat marginalized themes, “Motor Themes” representing well-developed and significant research areas, “Emerging or Declining Themes” indicating recently emerged or fading themes, and “Basic Themes” representing important yet incomplete themes. Keywords such as “depression,” “quality-of-life,” and “anxiety” fall between “Motor Themes” and “Niche Themes,” indicating that research in these directions had a solid disciplinary foundation but is currently somewhat marginalized. This underscored the potential for further enriching literature in these areas, signifying their importance and promising development trend. The simultaneous placement of keywords like “brain,” “mechanisms,” and “expression” between “Niche Themes” and “Emerging or Declining Themes” indicated a certain research foundation in these areas. However, accurately assessing developmental trends based solely on this chart may be challenging. On the other hand, keywords like “epilepsy,” “temporal-lobe epilepsy,” and “seizures” fall within the “Basic Themes” quadrant, affirming the foundational and essential nature of research in these directions. This suggested that researchers can gradually enhance exploration in these directions to provide a more comprehensive theoretical foundation for subsequent studies.

**Figure 7:**
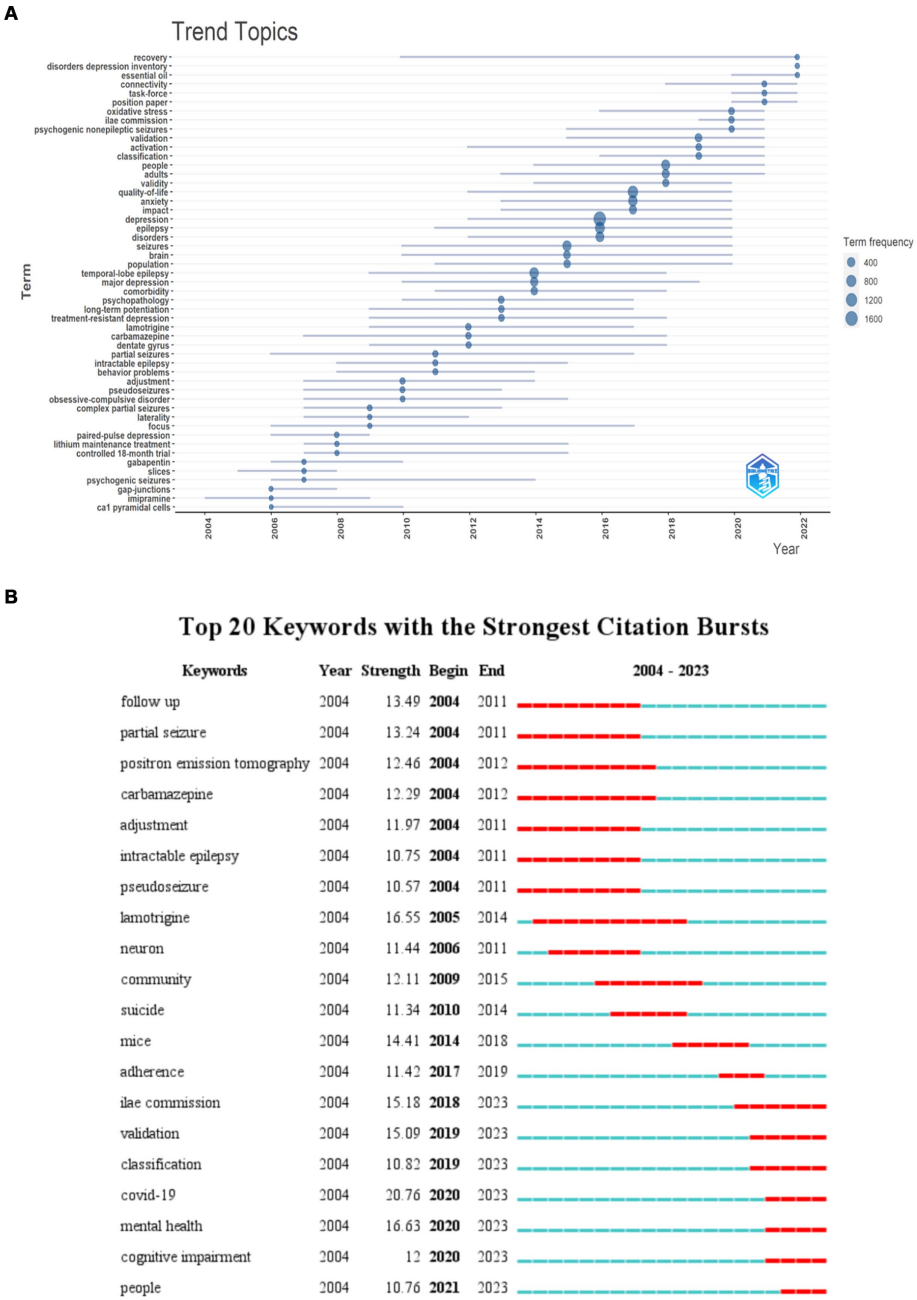
Keyword visualization. **(A)** Trend topics. Visualizations that occur more than 30 words per year. The nodes represent the years in which the words appear most frequently. **(B)** Keyword emergence map. The green timeline shows the duration of the relevant research, and the red timeline shows the emergence time of the relevant research.

Visual analysis of research hotspots in Figure 7A showed keyword hotspot predictions. Over the last 2 years (2022–2023), research association related to “recovery,” “disorders depression inventory” and “essential oil” have gained scholarly attention. This indicated that the field of disease recovery in comorbid epilepsy and depression is poised to emerge as a research hotspot in the forthcoming years.

Keyword emergence refers to words that appear with higher frequency in a specific period, effectively reflecting the trend of research topics in the field over the years. Figure 7B represented the top 20 keyword, with “lamotrigine” showing the longest emergence time from 2004 to 2014. Other keywords with significant emergence intensity include “follow up” (2003–2011), “partial seizure” (2004–2011), “positron emission tomography” (2004–2012), “carbamazepine” (2004–2012), and more. This illustrates the research hotspots in the comorbidity of epilepsy and depression from 2004 to 2012. In the last 3 years, keywords like “COVID-19” (2020–2023), “mental health” (2020–2023), “cognitive impairment” (2020–2023), and “people” (2021–2023) had shown significant emergence intensity. Beyond pandemic-related reasons, it indicated a growing interest in the topics like “mental health” and “cognitive impairment” in relation to comorbid epilepsy and depression, potentially emerging as future research hotspots.

### 3.5 Citation frequency analysis

“Most Global Cited Documents” are those that are cited in the Web of Science Core Collection (WoSCC) database, whereas “Most Local Cited Documents” are those that are cited in this collection of articles. Among the top 10 documents, only one publication, “Psychiatric comorbidity in epilepsy: A population-based analysis” by Tellez-Zenteno, JF in 2007, appears in both “Most Local Cited Documents” and “Most Global Cited Documents.” These results indicated significant influence in the research field of comorbid depression with epilepsy. We speculate that this influence is attributed to its population-based study evaluating the prevalence of psychiatric disorders in people with epilepsy (10), providing realistic estimates for the prevalence of various psychiatric disorders associated with epilepsy. Moreover, the study also offers essential information for the logical regression models of prevalence categorized by age and gender, further enhancing its significance and impact within the field.

## 4 Discussion

### 4.1 Main findings

In this study, we utilized the WoSCC database as the primary data source to analyze the literature on comorbid depression in epilepsy from 2003 to 2023, aiming to elucidate the research trends and hotspots in this area over the years. VOSviewer, CiteSpace and Bibliometrix were used to generate visualization knowledge maps, including annual trends of publications, contributions of countries, institutions, authors, journals, and clustering of keywords.

We found that the number of papers published in this field has shown an increasing trend in recent years, indicating that comorbid depression in epilepsy may continue to be a hot topic in the next few years. Furthermore, we observed that articles in this field primarily appear in the top four journals, namely *EPILEPSY & BEHAVIOR*, *EPILEPSIA, SEIZURE-EUROPEAN JOURNAL OF EPILEPSY* and *EPILEPSY RESEARCH*. Notably, *EPILEPSY & BEHAVIOR* not only leads in the number of publications but also in citation frequency. Additionally, our analysis of countries and publishing institutions indicates that the United States, United Kingdom, China, Germany, Italy, and Canada rank among the top six countries in terms of publication volume. The United States shows a dominant leading position and has the closest collaboration with the UK among all countries (Table 2).

**Table 2:** Cited frequency.

Local citations				Global citations		
Rank	Title	Journal	Frequency	Title	Journal	Frequency
1	Psychiatric comorbidity in epilepsy: A population-based analysis	EPILEPSIA	372	Evidence-based guidelines on the therapeutic use of repetitive transcranial magnetic stimulation (rTMS)	CLIN NEUROPHYSIOL	1,223
2	Depression but not seizure frequency predicts quality of life in treatment-resistant epilepsy	NEUROLOGY	321	Default-mode brain dysfunction in mental disorders: A systematic review	NEUROSCI BIOBEHAV R	1,177
3	Rapid detection of major depression in epilepsy: a multicentre study	LANCET NEUROL	319	Electrical stimulation of the anterior nucleus of thalamus for treatment of refractory epilepsy	EPILEPSIA	1,103
4	The relative impact of anxiety, depression, and clinical seizure features on health-related quality of life in epilepsy	EPILEPSIA	232	Evidence-based guidelines on the therapeutic use of transcranial direct current stimulation (tDCS)	CLIN NEUROPHYSIOL	886
5	Epilepsy and risk of suicide: a population-based case–control study	LANCET NEUROL	189	Taming THC: potential cannabis synergy and phytocannabinoid-terpenoid entourage effects	BRIT J PHARMACOL	861
6	The psychiatric comorbidity of epilepsy	ACTA NEUROL SCAND	180	Mental Health and Psychosocial Problems of Medical Health Workers during the COVID-19 Epidemic in China	PSYCHOTHER PSYCHOSOM	821
7	Epilepsy, suicidality, and psychiatric disorders: A bidirectional association	ANN NEUROL	166	Psychiatric comorbidity in epilepsy: A population-based analysis	EPILEPSIA	721
8	Depression in epilepsy A systematic review and meta-analysis	NEUROLOGY	165	Drug resistance in brain diseases and the role of drug efflux transporters	NAT REV NEUROSCI	683
9	Depression and comorbidity in community-based patients with epilepsy or asthma	NEUROLOGY	154	Steady-state visually evoked potentials: Focus on essential paradigms and future perspectives	PROG NEUROBIOL	680
10	Predictors of pharmacoresistant epilepsy	EPILEPSY RES	143	Evidence-Based Guideline: Treatment of Convulsive Status Epilepticus in Children and Adults: Report of the Guideline Committee of the American Epilepsy Society	EPILEPSY CURR	606

“Most Local Cited Documents” refers to the most cited documents in the exported data, indicating the impact in this field; “Most Global Cited Documents” refers to the most cited documents in the WOS database, indicating impact across all areas.

Institutional analysis identified key players such as the University of Melbourne, Columbia University, and King’s College London, predominantly situated in developed nations like the United States, the United Kingdom, and Canada. These findings clearly indicated that research on comorbid epilepsy and depression is mainly concentrated in developed countries.

Furthermore, authorship analysis identified significant contributors such as *Mula*, *Marco*, *Kanner*, *Andres M.*, and *O’Brien*, *Terence J*., who made substantial contributions in earlier years, alongside emerging figures like *Lee, Sang-Ahm* and *Sajatovic, Martha* who have made notable contributions in recent years, warranting sustained attention in future research endeavors.

Finally, our analysis of keyword co-occurrence reveals that the majority of research in this field is focused on clinical studies. There is limited research on the mechanisms underlying comorbid epilepsy and depression, with few studies utilizing experimental animal models in this context. Notably, keywords such as “brain” and “mechanisms” have become more prevalent in recent years, reflecting the emphasis on mechanistic research in this field.

### 4.2 Research hotspots related to epilepsy and depression

Given the limited understanding of the clinical pathogenesis of epilepsy comorbid with depression, most studies have focused on epidemiological investigations. By analyzing keywords such as “prevalence” and “population,” this study explores the research hotspots to elucidate the association between epilepsy and depression. Keywords like “suicide” and “follow-up” suggest that societal concerns about the mental health of this patient population drive research, revealing a bidirectional relationship between epilepsy and depression. A longitudinal cohort study using the UK General Practice Research Database compared the incidence of suicide-related mental disorders in individuals with and without epilepsy, demonstrating a bidirectional relationship between widespread mental disorders (i.e., depression, anxiety, psychosis) and epilepsy (11). Zhao et al. assessed the depression severity and suicide risk in 267 people with epilepsy, finding a positive correlation between the severity of depression and suicide risk (12). Additionally, Eva Bølling-Ladegaard’s team analyzed 36 years of data from Danish hospital registries, calculating hazard ratios for depression following an epilepsy diagnosis and vice versa, indicating an increased risk of both conditions (13) ^.^

Keywords such as “adults,” “adolescents,” and “children” indicate a broad age range for the comorbidity of epilepsy and depression. Research suggests significant age-related differences in the severity and symptoms of depression at different stages of epilepsy (14). Compared to childhood (15), adolescents with epilepsy have a higher risk of developing depression (16, 17). Given the less pronounced depressive symptoms in young children, the bidirectional relationship between epilepsy and depression is more evident during adolescence. However, current studies primarily focus on adult epilepsy, with a lack of research on children or adolescents, thus necessitating further exploration.

The field of research on epilepsy comorbid with depression and other comorbidities is currently underexplored. Our keyword co-occurrence analysis identified terms like “anxiety, ““AD” (Alzheimer’s disease), “PD” (Parkinson’s disease), and “migraine,” prompting investigation into the relationship between epilepsy, depression, and other comorbidities. Studies have found a strong comorbidity between anxiety and depression (18) suggesting susceptibility to anxiety disorders in addition to depression among people with epilepsy (19). Depression is also common in AD and PD (20, 21) with a bidirectional relationship between AD and epilepsy (22) and an increased risk of epilepsy associated with PD (23). Therefore, we hypothesize that there may be a bidirectional comorbidity relationship between depression in people with epilepsy and AD, PD. We hope that more research will be conducted to explore and discuss this hypothesis.

The recent surge in citations for the keyword “COVID-19” highlights the potential adverse impacts of large-scale public health events on the prevalence and severity of epilepsy comorbid with depression. During the COVID-19 pandemic, issues like unemployment, social isolation, economic difficulties, and difficulty accessing medication increased depression and anxiety levels among people with epilepsy, affecting their personal, social, and occupational lives (24). Research indicates that employment status, social support, and stigma are critical factors in assessing depression severity in people with epilepsy (25). Therefore, societal and governmental measures are advocated to protect their rights and interests during major public health crises.

### 4.3 Clinical therapies and drugs for comorbid epilepsy and depression

The keyword “diagnosis” reveals that clinical diagnosis of epilepsy comorbid with depression relies on screening scales such as the Neurological Disorders Depression Inventory for Epilepsy (NDDI-E), the Hospital Anxiety and Depression Scale Depression Subscale (HADS-D), and the Beck Depression Inventory (BDI) (26). Our keyword co-occurrence analysis identified terms like “antiepileptic drugs” and “lamotrigine,” indicating that many drugs originally intended for epilepsy are now used for treating comorbid depression due to their antidepressant effects. Research by Cramer JA et al. demonstrated that lamotrigine can improve depressive symptoms in people with epilepsy (27), making it suitable for treating comorbid depression. Evidence supports the association between serotonin (5-HT) and epilepsy (28, 29), showing that serotonin has an anticonvulsant effect in pilocarpine-induced epilepsy models, reducing seizure frequency. Depressive behaviors in this model are associated with decreased hippocampal serotonin, and serotonergic drugs like citalopram, imipramine, and fluoxetine can increase hippocampal serotonin release, exerting anticonvulsant effects and improving depressive behaviors. Therefore, serotonergic drugs can be used to treat epilepsy comorbid with depression (30). For people with epilepsy experiencing depressive episodes, selective serotonin reuptake inhibitors (SSRIs) are the first-line treatment. If monotherapy is ineffective, combining SSRIs with presynaptic autoreceptor inhibitors or adding lithium, quetiapine, and aripiprazole can enhance the antidepressant effect (31). Additionally, soluble epoxide hydrolase inhibitor TPPU and lacosamide have both anticonvulsant and antidepressant properties (32, 33).

Although pharmacotherapy is primary for alleviating symptoms of comorbid epilepsy and depression, keyword co-occurrence analysis also highlights “behavior,” “electrical stimulation,” and “EEG,” suggesting emerging therapies. Clinical trials have shown significant reductions in seizure frequency following cognitive behavioral therapy (CBT) (34) For refractory epilepsy and depression, vagus nerve stimulation (VNS) and transcranial direct current stimulation (tDCS) are main treatments (35), with tDCS improving depressive states in people with epilepsy. While non-pharmacological therapies are currently limited in scope and methodology (36), their research potential warrants attention and in-depth exploration.

### 4.4 Potential mechanisms of comorbid epilepsy and depression

Numerous studies have examined the relationship between epilepsy and depression, indicating an association (37, 38). Advancements in understanding the mechanisms of epilepsy comorbid with depression are crucial. As illustrated in Figure 6C, “mechanisms” are positioned between Niche Themes and Emerging or Declining Themes, suggesting high specialization but recent under-research. The scarcity of mechanism-related keywords highlights weaknesses in this area. Connections among epilepsy, depression, schizophrenia, Parkinson’s disease, and Alzheimer’s disease suggest that common mechanisms might provide insights into epilepsy comorbid with depression.

To guide future research, representative mechanism-related keywords from the top 200 most frequent keywords were extracted, including “inflammation,” “5-HT,” “GABA,” “stress,” “hippocampus” and “neuroregulation.” These keywords indicate that alterations in immune-related substances and disturbances in neurotransmitter balance are the predominant focus of current research.

The significance of neuroinflammation in the pathogenesis of epilepsy and depression is well-established (39). Shin et al. conducted ELISA detection of serum cytokines (IL-2β, IL-5, IL-6, IFN-γ, CCL134, and CCL32), revealing a potential association between psychiatric symptoms and the inflammatory process in people with epilepsy (40). Neuroglial cells play an important role in the onset of epilepsy, regulating inflammatory processes and neuronal excitability (41). Microglia may contribute to depression through the release of cytokines such as IL-1, IL-6, IL-8, and IFN8 (42). IL-8β can induce hyperfunction of the HPA axis and dysregulation of the 5-HT axis (43), potentially contributing to comorbid epilepsy and depression.

GABA is an inhibitory neurotransmitter with reduced activity in patients with primary major depression, potentially contributing to the disease (44, 45). Studies have revealed lower GABA tissue content in patients with temporal lobe epilepsy (TLE) combined with depression, significantly related to the frequency and duration of epileptic seizures (44).

Comorbid epilepsy and depression might also be stress-related. Under chronic stress, the hypothalamic–pituitary–adrenal cortex system (HPACS) becomes excessively activated, leading to the secretion of stress hormones like glucocorticoids into the bloodstream (46). Excessive glucocorticoids disrupt hippocampal function and impair its structure, including glucocorticoid receptor dysfunction, neurotransmitter imbalance, neurotrophic factor impairment, and neuroinflammation. This process may result in neurodegeneration and hippocampal neuron loss, forming the basis for comorbid epilepsy and depression (47). Dysregulation of the HPA axis has been observed in depression and chronic epilepsy animal models (48), potentially increasing the risk of comorbid depressive disorders in epilepsy (49).

Therefore, we speculate that regulating immune and inflammatory responses, increasing GABAergic activity, or reducing HPA axis activity may inhibit the occurrence or development of comorbid depression in epilepsy. It is necessary to further research and exploration to validate these biological mechanisms.

### 4.5 Advantages and limitations

This article presents the first bibliometric analysis specifically focused on comorbid depression in epilepsy, as identified through a comprehensive literature search. Given the increasing incidence rate of comorbid epilepsy and depression, this study holds significant implications for both clinical practice and academic discourse. The Web of Science (WoS) is among the most authoritative databases in the international academic community, with high international recognition. This study used the WoSCC database for literature retrieval, ensuring the quality of the selected articles; the research utilized three software tools for analysis: VOSviewer, CiteSpace and Bibliometrix. By employing rich visualizations and highlighting shifts in research focus over time, this study enables researchers to gain a comprehensive understanding of the foundational knowledge and evolutionary trajectory within the field over the past 20 years. Such insights facilitate the prediction of research hotspots and frontiers, thereby offering valuable reference points for future studies.

However, this study has certain limitations. Firstly, in terms of time, this study only conducted a literature analysis of the past 20 years, so the timeline is not complete; secondly, regarding database usage, only the literature on comorbid epilepsy and depression from the WoSCC database was included, which means the content of the selected literature is still limited; thirdly, English remains the preferred language of contemporary academic journals, hence our analysis only considered English literature, thereby excluding articles in other languages. Thus, future research endeavors should aim to overcome these limitations by employing a wider range of methodologies to thoroughly evaluate the content of this field and conducting more comprehensive and in-depth analyses.

## 5 Conclusion

In this paper, we conducted a comprehensive bibliometric and visual analysis of comorbid depression with epilepsy over the past two decades, including the authors, countries, publishing institutions and keywords, and made a prediction of future research hotspots in this field. The findings highlight that the journals *EPILEPSY&BEHAVIOR* and *EPILEPSIA* had the highest average citation times of literatures on comorbid epilepsy and depression, and were the authoritative journals in this field. The country with the largest number of publications is the United States, and the institution with the largest contribution to the number of publications is the University of Melbourne. Most authors in this field conduct research in the form of team cooperation. Through analysis, we find that the hot keywords in this field mainly focus on “recognitive impairment” and “mental health,” which we believe will be the potential research hotspots and trends in the future. This study aims to provide researchers with the current trends and information of the field and help them to grasp the hotspots.

## Data availability statement

The original contributions presented in the study are included in the article/supplementary material, further inquiries can be directed to the corresponding authors.

## Author contributions

G-YL: Conceptualization, Data curation, Formal analysis, Investigation, Methodology, Project administration, Resources, Software, Supervision, Validation, Visualization, Writing – original draft, Writing – review & editing. F-JF: Conceptualization, Data curation, Formal analysis, Methodology, Project administration, Software, Visualization, Writing – original draft, Writing – review & editing. Y-XC: Conceptualization, Data curation, Investigation, Software, Visualization, Writing – original draft. M-SY: Conceptualization, Data curation, Investigation, Methodology, Software, Visualization, Writing – original draft. Y-LO: Data curation, Formal analysis, Investigation, Methodology, Software, Visualization, Writing – original draft. M-DY: Data curation, Formal analysis, Investigation, Resources, Software, Visualization, Writing – original draft. LP: Data curation, Formal analysis, Investigation, Methodology, Software, Visualization, Writing – original draft. W-PL: Formal analysis, Funding acquisition, Project administration, Resources, Supervision, Writing – original draft, Writing – review & editing. WX: Funding acquisition, Project administration, Resources, Supervision, Validation, Writing – original draft, Writing – review & editing.
